# Microorganisms within Human Follicular Fluid: Effects on IVF

**DOI:** 10.1371/journal.pone.0059062

**Published:** 2013-03-12

**Authors:** Elise S. Pelzer, John A. Allan, Mary A. Waterhouse, Tara Ross, Kenneth W. Beagley, Christine L. Knox

**Affiliations:** 1 Institute of Health and Biomedical Innovation, Faculty of Science and Technology, Queensland University of Technology, Brisbane, Queensland, Australia; 2 The Wesley Research Institute, Women's Health Laboratory, The Wesley Hospital, Auchenflower, Queensland, Australia; 3 Wesley Monash IVF, The Wesley Hospital, Auchenflower, Queensland, Australia; University of Nevada School of Medicine, United States of America

## Abstract

Our previous study reported microorganisms in human follicular fluid. The objective of this study was to test human follicular fluid for the presence of microorganisms and to correlate these findings with the *in vitro* fertilization (IVF) outcomes. In this study, 263 paired follicular fluids and vaginal swabs were collected from women undergoing IVF cycles, with various causes for infertility, and were cultured to detect microorganisms. The cause of infertility and the IVF outcomes for each woman were correlated with the microorganisms detected within follicular fluid collected at the time of trans-vaginal oocyte retrieval. Microorganisms isolated from follicular fluids were classified as: (1) ‘colonizers’ if microorganisms were detected within the follicular fluid, but not within the vaginal swab (at the time of oocyte retrieval); or (2) ‘contaminants’ if microorganisms detected in the vagina at the time of oocyte retrieval were also detected within the follicular fluid. The presence of *Lactobacillus* spp. in ovarian follicular fluids was associated with embryo maturation and transfer. This study revealed microorganisms in follicular fluid itself and that the presence of particular microorganisms has an adverse affect on IVF outcomes as seen by an overall decrease in embryo transfer rates and pregnancy rates in both fertile and infertile women, and live birth rates in women with idiopathic infertility. Follicular fluid microorganisms are a potential cause of adverse pregnancy outcomes in IVF in both infertile women and in fertile women with infertile male partners.

## Introduction

The presence of opportunistic pathogens in the lower female reproductive tract has been associated with adverse pregnancy outcomes after both natural and IVF conceptions [1] and we have also demonstrated differences in women with colonized and contaminated follicular fluid [2]. In addition, a number of studies have confirmed that microorganisms frequently and transiently colonize the female upper genital tract in the absence of a symptomatic infection [3], [4], [5]. Studies investigating microorganisms and human follicular fluid have been mainly undertaken in women participating in IVF cycles because of the nature of the procedures required to obtain this specimen [2],[6],[7].

In their study, Cottell *et al* [6] analyzed the effect of microorganisms from the IVF culture system as a whole by pooling the results obtained for each specimen type (follicular fluid, oocyte retrieval needle washes, semen and culture media) and seeking associations between these results and IVF outcomes and concluded that there were no detrimental effects. Cottell also reported a significant decrease in the number of oocytes retrieved from women when microorganisms were isolated from their follicular fluid.

The aim of this current study was to screen follicular fluid collected from IVF patients at the time of trans-vaginal oocyte retrieval for the presence of microorganisms. We hypothesized that follicular fluid microorganisms cause adverse IVF outcomes. Therefore, we identified the microorganisms in follicular fluids and vaginal swabs and correlated these with IVF outcomes.

## Materials and Methods

### Specimen collection

From September 2007 to November 2008, 263 consenting couples commencing fully stimulated IVF cycles at Wesley-Monash IVF (Brisbane, QLD) were enrolled in this study. Pregnancy was classified by the World Health Organization definition of a clinical pregnancy with fetal heartbeat: that is ultrasonographic evidence of a gestational sac containing one or more fetuses with a heart beat [8].

Women from couples where the only cause of infertility was male factor infertility were classified as the ‘fertile’ control group. The remaining women were grouped according to an etiology of infertility due to: (1) endometriosis, which was laparoscopically diagnosed by identification of endometrial explants within the pelvic cavity; (2) polycystic ovary syndrome, diagnosed by the Rotterdam criteria [9]; (3) a history of genital tract infection, defined by positive testing for *Chlamydia trachomatis* in either partner, pelvic inflammatory disease, sexually transmitted infection, or laparoscopic diagnosis of tubal disease; or (4) idiopathic infertility if the female and male partners had undergone screening for infertility and no abnormalities were detected. Couples were enrolled in this study in the order in which they consented and began IVF treatment. No patients were prescribed antimicrobial therapy during the course of their IVF cycle. No efforts were made to control the distribution of group sizes based on the etiology of infertility.

Specimen collection and storage was performed as previously described [2]. Two types of specimens were collected from each woman: follicular fluid samples from the left and right ovary where available (n = 463), and vaginal swab specimens (n = 263), which were cultured for the detection and identification of microbial species present. The follicular fluid specimens were aseptically transferred to a sterile culture dish to determine if there was an oocyte present. Following removal of the oocyte, the IVF scientists transferred the remaining follicular fluid to a sterile 15 mL Falcon tube for storage at −80°C. The vaginal swabs were collected prior to trans-vaginal oocyte retrieval and following the preparation of the vagina with sterile water. Preparation of the vaginal wall is performed to remove cell debris and mucous, rather than microorganisms. The vaginal swabs were collected following the vaginal preparation to ensure that the only species recovered were those remaining when the needle passed through the vaginal wall at the time of follicle aspiration.

### Ethics statement

Ethical approval was obtained from the review boards of Uniting Care Health, Human Research Ethics Committee and Queensland University of Technology Human Ethics Committee. All patients provided informed written consent for their follicular fluids to be used in this study and gave permission for researchers to access medical records to obtain their reproductive history and IVF outcomes. All patients gave permission for researchers to access medical records to obtain their reproductive history and IVF outcomes.

### Isolation and identification of microorganisms from follicular fluid and vaginal swabs

Culture and identification of clinical isolates, was performed as previously described [2]. Briefly, differential and selective media were inoculated with 1 µL or 10 µL of follicular fluid. Semi-quantification of the colonies was performed by counting the approximate numbers of colonies on the Horse blood agar plate incubated aerobically or anaerobically and multiplying that number by the dilution factor appropriate for the inoculating loop used (10^3^ for a 1 µL loop or 10^2^ for a 10 µL loop) to give the colony forming units (CFU)/mL. The limit of detection using this method is 10^3^ CFU/mL.

### Statistical analysis

Statistical analyses were performed on the microbiological data and IVF outcomes using SPSS version 17 for Windows XP and R (R Development Core Team, 2011). Proportions were compared using a normal approximation or Fisher's exact test. Continuous variables were compared across groups using Wilcoxon's rank sum or the Kruskal-Wallis test. Multinomial logistic regression and Fisher's exact test were used to model the relationship between categorical outcomes (fertilization, embryo discard, embryo transfer, pregnancy) and explanatory variables (colonizing or contaminating species). A p-value of p<0.05 was considered statistically significant.

## Results

### Experimental design and patient demographics

Clinical specimens were collected from 263 women for microbiological analyses. One woman had no microorganisms detected in her follicular fluid by either traditional culture or molecular microbiology techniques, and her IVF outcome results were excluded from further analysis. For the remaining 262 women, the microorganisms identified in follicular fluid were analyzed to determine if there was a relationship between the microbial flora and IVF outcomes.

The mean age of women in this study was 37 years ±4 years. There were no statistical differences in the mean age of women from ‘fertile’ women with male factor infertility (37±4 years) compared to infertile women (37±6 years, p>0.05). There was no relationship between the age of the women who had a live birth (34±4 years) compared to those women who did not (33±4 years, p>0.05). For fertile and infertile women there was no difference in the number of prior IVF treatment cycles. Fertile women participated in an average of 1 (range 0–4±1 cycle) previous trans-vaginal oocyte retrieval procedure as did infertile women (range 0–5±1 cycle, p>0.05). There was no difference in the number of oocytes collected at the time of retrieval for fertile (11±6) or infertile women (11±6, p>0.05).

### Culture and colony identification of species isolated from follicular fluid and vaginal secretion

**Figure 1:**
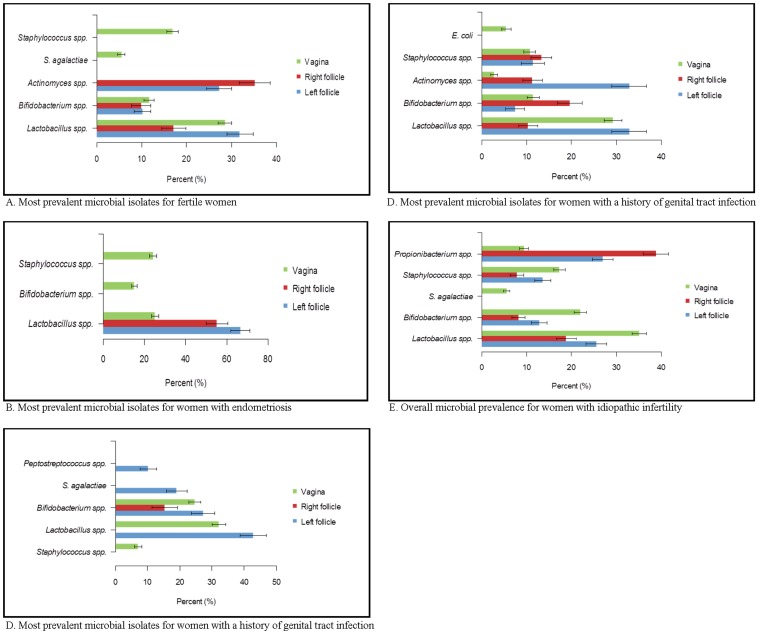
The most prevalent microbial species detected from the left and right follicular fluids and vagina from women in this study. 1 A–E The most prevalent microbial species detected from the left and right follicular fluids and vagina from women in this study are stratified based on the diagnosed cause of infertility. Bars represent one standard error.

Microorganisms were detected within 100% of cultured vaginal swabs collected after preparation of the vaginal vault for trans-vaginal oocyte retrieval. *Lactobacillus* spp., *Bifidobacterium* spp. and *Staphylococcus* spp. were the most prevalent species detected in the lower genital tract specimens of all women. The culture analyses revealed that cultivable bacterial species (1–5 species) were present in 99% of follicular fluids tested. A range of microorganisms was isolated from the follicular fluid. The follicular fluids were classified as: (1) ‘colonized’ (75/262, 29%) if microorganisms were detected within the follicular fluid, but not in the vagina (at the time of oocyte retrieval); or (2) ‘contaminated’ (187/262, 71%) if microorganisms detected in the vagina at the time of trans-vaginal oocyte retrieval were also detected within the follicular fluid. Both fertile women (those who have an infertile male partner; n = 60) and infertile women (n = 202; various causes of infertility) demonstrated both colonization and contamination of follicular fluid specimens (Table 1). The percentage of fertile women with colonized follicular fluids was 27%. The rates of colonization in infertile women ranged from 24% to 37% with no evidence of a difference in colonization rates across cohorts based on the causes of infertility: infertile women with endometriosis, polycystic ovary syndrome, or a history of genital tract infection (p>0.05). Culture analyses revealed that between zero and five different microbial species were detected within the left follicular fluid specimens and the right follicular fluid specimens Fewer follicular fluids from the left ovary contained no detectable microorganisms (p = 0.004) or two different microbial species (p = 0.007) when compared to right ovarian follicular fluids. More left ovarian follicular fluid samples contained four detectable microbial species (p<0.0001) compared to fluids collected from the right ovary. p-values compare results for left and right follicles.. There was a significant difference in the median number of microorganisms isolated from the left (n = 3) and right (n = 2) follicular fluids (p<0.0001) (Results not shown). Culture of vaginal swabs detected 1–11 different microbial species. There were subtle differences in the most prevalent microbial species detected in the left and right follicular fluids and in the vaginal swabs of women with colonized and contaminated follicular fluid (Figure 1 A–E). *Lactobacillus* spp. and *Actinomyces* spp. were also among the most prevalent species isolated from the upper genital tract of fertile women and women with a history of genital tract infection (Figure 1 A and D) except in women diagnosed with idiopathic infertility.

**Table 1:** Number of women with colonized or contaminated follicular fluid and the causes of infertility.

	Infertile women				Fertile women	
	n = 202				n = 60	
Etiology of infertility	Endometriosis	Polycystic ovary syndrome	Genital infection	Idiopathic	Male factor^1^	Total
	(n = 49)	(n = 48)	(n = 39)	(n = 66)	(n = 60)	(n = 262)
Colonized follicular fluid	18 (37%)	14 (29%)	11 (28%)	16 (24%)	16 (27%)	75 (29%)
Contaminated follicular fluid	31 (63%)	34 (71%)	28 (72%)	50 (76%)	44 (73%)	187 (71%)

1 ‘Fertile’ women with infertile male partners.

**Figure 2:**
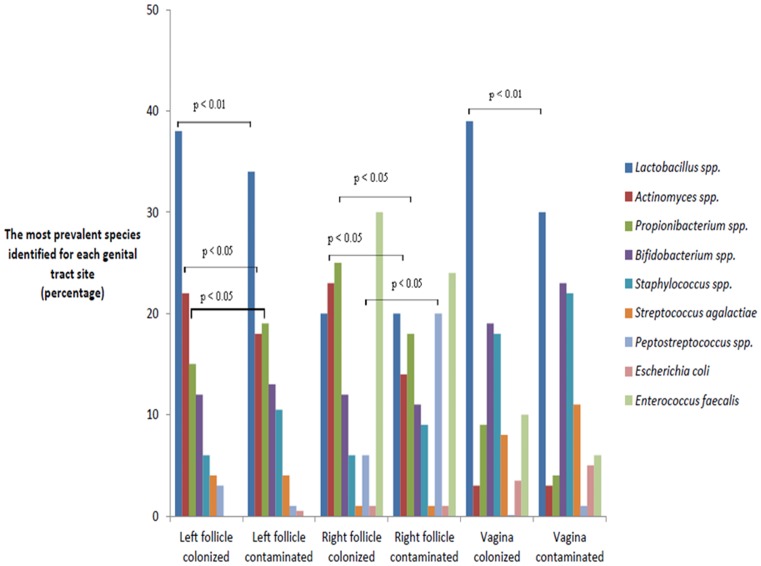
The most prevalent microbial species detected within the left or right follicular fluid and vagina in women with colonized or contaminated follicular fluid. *Lactobacillus* spp., *Actinomyces* spp. and *Propionibacterium* spp. were detected more often in the colonized left follicular fluids than in the contaminated left follicular fluids (p<0.01, p<0.05 and p<0.05 respectively). *Actinomyces* spp. and *Propionibacterium* spp. were identified more often in colonized than in contaminated follicular fluids (p<0.05), while *Peptostreptococcus* spp. were isolated more frequently from contaminated follicular fluids from the right ovarian follicles (p<0.05). The vaginal swabs from women with colonized follicular fluid had a higher number of *Lactobacillus* spp. isolated (p<0.01) and also a higher number bacterial isolates when compared to women with contaminated follicular fluid specimens (p<0.05); however, the same species were detected in both groups of women.

In left follicular fluids, *Lactobacillus* spp. and *Actinomyces* spp. were isolated more often from colonized than from contaminated follicular fluids (p<0.01 and p<0.05 respectively) (Figure 2). In the follicular fluids collected from the right ovary, *Actinomyces* spp. and *Propionibacterium* spp. were the only species isolated significantly more often from the colonized fluid than from contaminated follicular fluids (p<0.05). Also, *Peptostreptococccus* spp. was isolated more frequently from right contaminated follicular fluid than from colonized follicular fluid (p<0.05).The *Lactobacillus* spp., *Bifidobacterium* spp., *Staphylococcus* spp., and *S*. *agalactiae* were the most prevalent species identified as vaginal flora (Figure 1 A). *C*. *albicans*, *S*. *viridans*, *Fusobacterium* spp., and *L*. *iners* were detected only as follicular fluid contaminants, but not as follicular fluid colonizers (Table S1). None of these individual species was associated with either the etiology of infertility or the IVF outcomes (p>0.05).

### Colonized follicular fluid and the etiology of infertility

Logistic regression analysis was used to test for the degree of dependence between colonization of follicular fluid (either left or right) and etiology of infertility. Endometriosis, polycystic ovary syndrome, genital tract infection, male factor and idiopathic etiologies were considered. Colonization of left follicular fluid and endometriosis were found to be associated (p<0.05). There were no other statistically significant results.

### The etiology of infertility and IVF outcomes

Overall, for women with microbial colonization of follicular fluid there was no significant difference between fertilization rates for infertile women with a diagnosis of endometriosis, polycystic ovary syndrome or a history of upper genital tract infection when normalized against the control group of fertile women (Table 2).

**Table 2:** A comparison of IVF outcomes for women with different causes of infertility and colonized or contaminated follicular fluid.

	IVF outcomes														
	Fertilization rate^1^			Embryo discard rate^2^			Embryo transfer rates^3^			Pregnancy rate^4^			Live birth rate^5^		
Etiology	Contaminated follicular fluid	Colonized follicular fluid	p-value	Contaminated follicular fluid	Colonized follicular fluid	p-value	Contaminated follicular fluid	Colonized follicular fluid	p-value	Contaminated follicular fluid	Colonized follicular fluid	p-value	Contaminated follicular fluid	Colonized follicular fluid	p-value
Endometriosis (n = 49)	182/312 (58%)	122/196 (62%)	>0.05	62/182 (34%)	77/122 (63%)	<0.0001	29/31 (94%)	7/18 (39%)	< 0.0001	16/29 (55%)	0/7 (0%)	0.011	12/29 (41%)	0/7 (0%)	>0.05
Polycystic ovary syndrome (n = 48)	294/439 (67%)	142/217(65%)	>0.05	100/294 (34%)	52/142 (37%)	>0.05	27/34 (79%)	5/14 (36%)	0.006	16/27 (59%)	0/5 (0%)	0.043	8/27 (30)	0/5 (0%)	>0.05
Genital infection (n = 39)	203/329 (62%)	109/159 (69%)	>0.05	64/203 (32%)	36/109 (33%)	>0.05	19/28 (68%)	7/11 (64%)	>0.05	10/19 (53%)	1/7 (14%)	>0.05	5/19 (26%)	1/7 (14%)	>0.05
Idiopathic (n = 66)	336/497 (68%)	95/150 (63%)	>0.05	138/336 (41%)	39/95 (41%)	>0.05	36/50 (72%)	12/16 (72%)	>0.05	10/36 (28%)	7/12 (58%)	>0.05	6/36 (17%)	6/12(50%)	0.048
Male factor^6^ (n = 60)	291/520 (56%)	102/179 (57%)	>0.05	134/291 (46%)	43/102 (42%)	>0.05	33/44 (75%)	9/16 (56%)	>0.05	16/33 (48%)	0/9 (0%)	0.008	8/33 (24%)	0/9 (0%)	>0.05

1 No. of oocytes fertilized/total oocytes collected for all women in this cohort, ^2^No. of embryos discarded due to developmental arrest or degeneration from the total number of embryos produced by *in vitro* fertilization, ^3^The number of embryo transfers performed on the women within this cohort, not all women had an embryo transfer, ^4^The number of pregnancies resulting after embryo transfer, ^5^Live birth rate per embryo transfer, ^6^‘Fertile’ women control group – women undergoing IVF treatment as their male partners are infertile.

Adverse IVF outcomes were associated with microbial colonization of follicular fluid. Embryo discard rates were higher for women with endometriosis with colonized follicular fluid (63%, p<0.0001) but not for women with contaminated follicular fluid (34%) (Table 2). Decreased embryo transfer rates were observed in women with colonized follicular fluid (women with endometriosis, 39%, p<0.0001 and women with polycystic ovary syndrome, 36%, p<0.0001) when compared to women with contaminated follicular fluid (94% and 79% respectively) (Table 2). Decreased pregnancy rates were also observed for women with microbial colonization of follicular fluid (fertile women, 0%, p = 0.008; women with infertility due to endometriosis, 0%, p = 0.011; women with polycystic ovary syndrome, 0%, p = 0.043) when compared to women with contaminated follicular fluid (48%, 55% and 59% respectively). The etiology of infertility was not associated with decreased pregnancy rates for infertile women with a history of genital tract infection (14%) or idiopathic infertility (58%) when compared to the fertile women (p>0.05) (Table 2). Overall, colonized follicular fluid was associated with a decrease in the embryo transfer and pregnancy rates for all women (fertile and infertile – all four infertile groups combined) (p<0.05). The live birth rates were decreased for women with idiopathic infertility and contaminated follicular fluids (17%, p = 0.048) when compared to women with colonized follicular fluids (50%).

### Microbial colonization of follicular fluid and IVF outcomes

No single species was associated with decreased fertilization rates; however, the presence of *Lactobacillus* spp. in both right and left follicles was associated with higher rates of embryo transfer (p<0.05). By contrast, the presence of *Propionibacterium* spp. and *Streptococcus* spp. in right follicles was associated with poor embryo transfer rates (p<0.05 and p<0.01 respectively). *Propionibacterium* spp. were more frequently isolated from colonized right ovarian follicular fluids, whilst *S*. *agalactiae* was isolated more frequently from the left ovarian follicular fluids.

**Figure 3:**
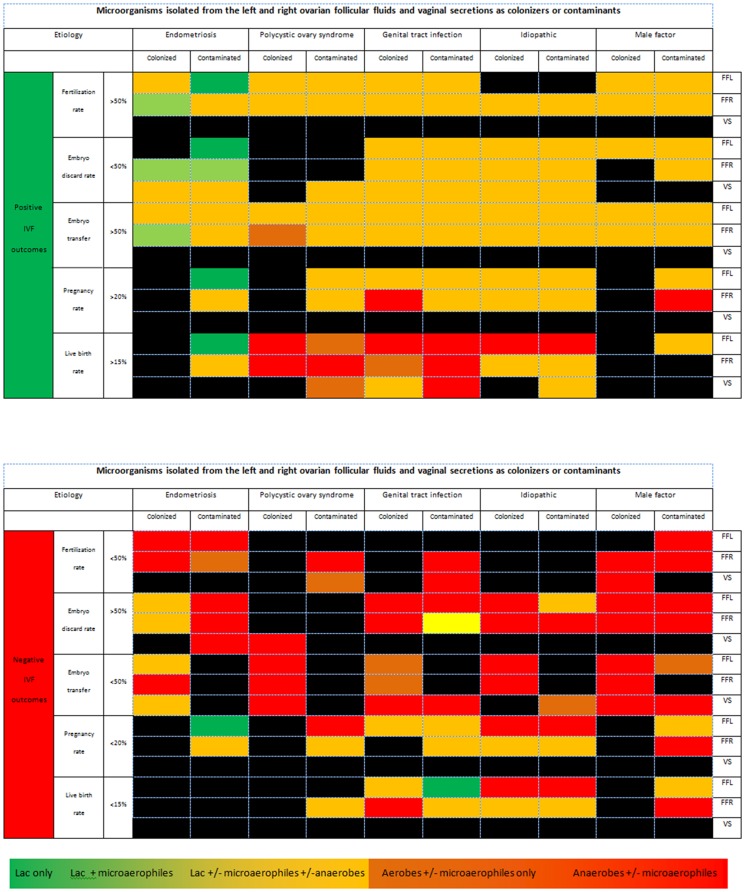
Heat map depicting IVF outcomes for fertile and infertile women with microorganisms detected from genital tract secretions during IVF cycles. Standard microbiological culture techniques were used to isolate and identify resident microorganisms from clinical specimens obtained from 262 women. Data represents the results obtained from culturing 262 vaginal swabs and 462 follicular fluids. All specimens were cultured on/in a range of microbiological culture media and broth. The heat map consists of a graphical two-dimensional matrix of colored squares, where each square represents the combined observations of the microorganisms isolated from all samples in each etiological cohort. The color of the square is dependent on the composition of the microorganisms at each anatomical site (FFL – left follicular fluid, FFR – right follicular fluid, VS – vaginal swab). Dark green squares represent specimens where only *Lactobacillus* spp. were isolated, light green squares represent specimens elaborating lactobacilli plus aerobic bacteria and/or microaerophilic bacteria. Light orange squares were used when *Lactobacillis* spp. were isolated alongside aerobic bacteria and/or microaerophilic bacteria, and/or anaerobic bacteria. Dark orange shading was used when only aerobic bacteria and/or microaerophilic bacteria were present but lactobacilli were absent and finally, red shading was used only if anaerobic species were the sole microbial isolates from the clinical specimen. The squares were ordered firstly by additional categorical data including the cause of infertility, as well as whether the microorganisms were defined as colonizers or contaminants at each site, and secondly by the IVF outcomes for fertilization rate, embryo discard rate, embryo transfer rate, pregnancy rate and live birth rate on the two axes. The combination of the three datasets into the one matrix allowed the cause of infertility, the resident microorganisms and the IVF outcomes to be visually examined and related to one another. Figure 3A represents the positive IVF outcomes and has an overall cooler appearance (green – light orange), whilst Figure 3B represents the negative IVF outcomes and has an overall warmer appearance, being predominantly dark orange to red. In black shaded squares, no relationship was evident between the microbial groupings, the cause of infertility, and the IVF outcomes.

There was also an association between pregnancy outcomes and different species in either the left or the right ovarian follicular fluid specimens. The presence of *Actinomyces* spp., *Bifidobacterium* spp., *Propionibacterium* spp. and *Streptococcus* spp. within the left ovarian follicular fluid had a significant association with negative pregnancy outcomes (p<0.01). *Lactobacillus* spp. as a contaminant within the left ovary was associated with a positive pregnancy outcome (p<0.001). For the right ovary, negative pregnancy outcomes were associated with *Actinomyces* spp., *Bifidobacterium* spp., and *Streptococcus intermedius* (p<0.01), and to a lesser extent with *Propionibacterium* spp. and *Staphylococcus* spp. (p<0.05). To demonstrate the relationship between individual or combination microbial species and IVF outcomes in this study, combinations of microbial species were color coordinated based on known genital tract pathogenicity for presentation as a heat map (Figure 3). For example, the lactobacilli are associated with a positive role in the genital tract and therefore, when isolated alone were colored dark green; however, the presence of other microbial species can reduce the protective nature of the lactobacilli, reflected by light green and orange shading [10], [11], [12], [13], [14] (Table 3).

**Table 3:** Heat map key to shading based on microbial composition within the sample.

Microorganism isolated	Dark green	Light green	Light orange	Dark orange	Red
*Lactobacillus* spp.	√	√	√	×	×
Any aerobe or microaerophile	×	+/−	√	√	+/−
Any anaerobe	×	×	+/−	×	√

We have presented the heat map as the IVF outcomes plotted against each cause of infertility. IVF outcomes have been arbitrarily grouped as positive (fertilization rate >50%, embryo discard rate <50%, embryo transfer rate >50%, pregnancy rate >20%, live birth rate >15%) and negative (fertilization rate <50%, embryo discard rate >50%, embryo transfer rate <50%, pregnancy rate <20%, live birth rate <15%). The heat map demonstrates that the lactobacilli are more frequently isolated alone or in combination with microaerophiles or anaerobes when a positive IVF outcome was achieved. In contrast, there is an absence of lactobacilli when poor IVF outcomes were reported. As the map is viewed from top (positive IVF outcomes) to bottom (negative IVF outcomes) there is a transition from green through to red, demonstrating the particularly negative impact of isolating anaerobes in the absence of *Lactobacillus* spp. Statistical analyses on all individual species were not appropriate due to the small number of observations reported for some; however, the heat map does correlate with findings reported for species with sufficient observations, and provides additional information pertaining to the remaining species isolated from the genital tract specimens of women undergoing IVF cycles.

## Discussion

This study found that human follicular fluid contains microorganisms. The presence of some microorganisms (*Propionibacterium* spp., *Streptococcus* spp. *Actinomyces* spp., *Staphylococcus* spp. and *Bifidobacterium* spp.) correlated with adverse IVF outcomes whereas improved embryo transfer rates are associated with the presence of *Lactobacillus* spp. in both the left and right ovarian follicular fluids. Our data demonstrates that not only is follicular fluid not sterile but that bacterial species in follicular fluid can have both positive and negative effects on IVF outcomes.

Previous studies have also reported the presence of bacteria within porcine follicular fluid. These studies concluded that some bacteria (*E. coli* and *Streptococcus* spp.) in porcine follicular fluid might inhibit follicle-stimulating hormone (FSH) from binding to its receptor on granulosa cells [15], [16], [17]. In the ovary, the FSH receptor is essential for follicular development and oocyte maturation. Such inhibition would prevent the normal hormonal functioning of FSH. It is therefore plausible that the presence of microorganisms in human follicular fluid may result in inhibition of the functioning of FSH, damage to the cumulus oocyte complex, the subsequent immune response within the follicular fluid during folliculogenesis or in the uterus at the time of implantation either by the microorganisms themselves, or the microbial products of metabolism. Identification of bacteria colonizing the follicular fluid in couples experiencing a prolonged failure to conceive may present the clinician with an opportunity to initiate antimicrobial treatment prior to the next attempt at conception [18], [19]. It may also provide a reason for failure to conceive in the absence of another explanation.

IVF embryo transfer rates and pregnancy rates showed a dependence on the microbial species colonizing the ovary. Further investigations are required to determine whether these genera affect the embryo quality and thus contribute to the complex physiological processes involved in failed implantation or early pregnancy loss.

Improved embryo transfer rates are associated with the presence of *Lactobacillus* spp. in both the left and right ovarian follicular fluids. The degree of correlation of the results differed between the left and right ovaries, and we propose that this may be due to the presence of different lactobacilli colonizing each ovary (*L*. *crispatus* and *L*. *gasseri*, left ovary; *L*. *iners*, right ovary) (Table S1). Each species of lactobacilli differs in its ability to produce hydrogen peroxide and lactic acid, which are considered to be inhibitory substances to other microbial species [20], [21], [22]. It is interesting that in previous studies of women undergoing IVF treatment cycles, the culture of the catheter tips from embryo transfers revealed that the presence of hydrogen peroxide producing *Lactobacillus* spp. from the vagina/endometrium was associated with improved embryo transfer rates and successful IVF pregnancy outcomes (Moore *et al*., 2000). However, the presence of bacteria other than *Lactobacillus* spp. (including *Escherichia coli*, *Streptococcus* spp., other *Enterobacteriaceae*, *Staphylococcus* spp., *Haemophilus* spp. and mixed cultures), reported as cervicovaginal flora, were associated with a reduced number of successful IVF pregnancies and an increase in early miscarriage rates [12], [13], [14], [23], [24]. No microbiological specimens from the upper genital tract were collected and/or screened in these prior studies. It may therefore be possible that the microorganisms detected on the catheter tips used for transferring the embryos to the uterus, originated from the follicular fluid entering the upper genital tract/peritoneal cavity at the time of trans-vaginal oocyte retrieval. These microorganisms have the potential to persist within the female upper genital tract and to elicit an inflammatory immune response, which could adversely affect pre and post conception outcomes in the current or future cycles [10], [25], [26], [27] Are microorganisms in human follicular fluid already present, or are they introduced during IVF procedures? Microorganisms that were not simultaneously present within the lower genital tract as normal regional flora were detected in follicular fluid, suggesting that the microorganisms are not always introduced as part of the IVF procedure, but may colonize the fluid independently. Potential sources of follicular fluid microorganisms, spread to the fluid via hematogenous dissemination include the oral mucosa and respiratory tract [28], [29], [30], [31]. We note that due to the collection technique, some samples classified as contaminated in this current study might actually be colonized samples, where the follicular fluids were independently colonized with the same microorganisms as the vagina.

In agreement with our findings, previous studies have also reported discordant results when investigating the microorganisms isolated from the lower and upper genital tracts of asymptomatic women. *Bacteroides* spp. and *Streptococcus* spp. have been recovered from peritoneal fluid, but not from the vagina or cervix for 25% of women tested [3]. This suggested that bacteria may exist in the upper genital tract without any evidence of infection. Asymptomatic or symptomatic colonization or infection of the upper genital tract in non-pregnant women has been reported [3], [4], [32]. The female upper genital tract, once considered ‘normally sterile’, has been shown to harbor many potentially pathogenic microorganisms, in non-pregnant women without inflammatory disorders [5]. Further, a recent molecular based study also identified bacteria in suprapubic urine aspirates from the bladders of asymptomatic women [33]. In our study, frequently encountered species found to be colonizing follicular fluids were members of the normal regional flora of the vagina (*Lactobacillus* spp.), the gastrointestinal tract (*Bifidobacterium* spp., enteric bacteria, *S*. *agalactiae*), the skin (*Staphylococcus* spp.) and the oral mucosa (*Streptococcus* spp.) (Table S1). This further supports the idea that the follicular fluid is not always contaminated as part of the IVF oocyte retrieval process, but rather, may be colonized independently.

We employed a culture-dependent method for the primary isolation and identification of the microorganisms present within follicular fluids and vaginal swabs. We confirmed that the *Lactobacillus* spp. were the most prevalent species isolated from these specimens in women with colonized and contaminated follicular fluids. This is in agreement with previous studies investigating the vaginal microflora in women undergoing IVF cycles, which also concluded that the lactobacilli are the most prevalent isolates [11], [34], [35]. More recent studies in this fieldemploy both culture and culture-independent methods to characterize the vaginal microbiota, and demonstrate even greater diversity than reported in our study.

The species of microorganisms we isolated from follicular fluid were consistent with previous reports documenting asymptomatic contamination of follicular fluid collected as part of an IVF cycle [6]. This current study extends the knowledge in the field by also identifying colonizing microorganisms within the follicular fluid. Analysis of the Cottell *et al* [6] data when the microorganisms were re-classified as ‘colonizers’ or ‘contaminants’ (based on the definition provided in our study) resulted in a pregnancy rate of 10% if follicular fluid microorganisms were contaminants and only 3% if the microorganisms were defined as colonizers. A larger sample size may have revealed associations between follicular fluid microorganisms and IVF outcomes. The identification of colonizing species in follicular fluids from both fertile and infertile women suggests that at least some microorganisms in follicular fluid may form part of the body's natural microflora. Alterations in follicular fluid microflora may be related to reproductive immunopathologies associated with endometriosis, polycystic ovary syndrome or tissue damage associated with prior inflammation and/or infection. The trend towards increased numbers of follicular fluid colonizers in women with endometriosis may be due to the underlying disease pathology, which requires the development of an extensive vasculature to support explanted endometrial tissue. Ovarian endometriomas occur in 17–44% of women with endometriosis [36], perhaps suggesting that the uterus itself may be the source of microorganisms in the ovary due to transplantation of colonized endometrial tissue. It has been suggested that the clockwise flow of the peritoneal fluid and the hydrostatic pressure within the abdominal cavity, which causes static pooling of fluid near the sigmoid colon, is a major contributing factor to the asymmetry of pelvic and abdominal pathology [37]. Therefore, the increased prevalence of ovarian endometriomas in the left ovary compared to the right [38], [39], [40] might also explain the increased prevalence of microorganisms we reported for follicular fluids collected from the left side.

The follicular fluid microorganisms were discordant for fluids collected from the left and the right ovaries. This study identified a significant difference in the median number of microorganisms isolated from the left compared to the right follicular fluids. This study confirms the results we reported in our smaller microbiological study (n = 14 microbial species were detected in colonized follicular fluid; n = 33 in contaminated follicular fluid, collected from the left ovary versus n = 7 and n = 21 respectively for fluids collected from the right ovary) [2]. Cottell *et al* [6] also reported a greater prevalence of cultivable microorganisms in left follicular fluids compared to right follicular fluids (42% left and 32% right). The blood supply to the ovary may facilitate the hematogenous transport of microorganisms from other anatomical sites. The vasculature to each ovary is asymmetrical and it has been reported that drainage of the left ovary is slower than the right because the left drains into the renal vein and then the inferior vena cava, whilst the right ovary drains directly into the vena cava [41]. The slower drainage of blood from the left ovary may mean that microorganisms present at the site persist for a longer period than those in the right ovary leading to poorer quality oocytes and early embryonic demise. The asymmetrical vascularization of the ovaries also supports the variation we have identified in the microbial colonization of left and right follicular fluids.

It has been proposed that ovulation occurs more often in the right ovary than the left ovary throughout the reproductive lifetime because of the separate, independent vasculature of each ovary [42], [43], [44]. One study reported significantly better IVF outcomes for oocytes from the right ovary; with an increase in the number of oocytes retrieved, and the fertilization rate compared to those from the left ovary [45]. A greater number of high quality embryos also developed from fertilized oocytes collected from the right ovary. However, the pregnancy and implantation rates were similar for transferred good quality embryos created from either ovary, suggesting that oocyte and early embryo quality are most affected by the *in vivo* follicular environment. Poor quality embryos will have been discarded following developmental arrest prior to transfer. Inequality in function between the left and right ovaries has also been reported in other mammals [46].

The data from this study demonstrate several new findings: (1) some species isolated from follicular fluid appear to be opportunistic pathogens (including *Propionibacterium* spp., *Streptococcus* spp. *Actinomyces* spp., *Staphylococcus* spp. and *Bifidobacterium* spp.) and are related to adverse IVF outcomes including decreased embryo transfer rates, which may reflect poor quality embryos due to damage by microorganisms or their metabolites and decreased pregnancy rates or no pregnancy (failed implantation); (2) *Lactobacillus* spp. within follicular fluid correlates with positive IVF outcomes; (3) follicular fluid is not sterile, but in fact contains a diverse range of microorganisms at varying concentrations; (4) follicular fluid can be colonized or contaminated by microorganisms; (5) follicular fluid colonization or contamination does not correlate with any one cause of infertility; and (6) the microflora of the follicular fluid is asymmetrical with differences observed between the microflora collected from the left and from the right ovary and in the IVF outcomes for each side.

The presence of microorganisms in follicular fluid may be a significant contributor to adverse IVF outcomes. We have shown that ovarian follicles can be colonized, and that the presence of microorganisms in human follicular fluid collected at the time of trans-vaginal oocyte retrieval for IVF can result in poor IVF outcomes affecting embryo quality, pregnancy rates and ultimately the live birth rate. Our results suggest that women with repeated failed IVF cycles may benefit from microbial screening of vaginal swabs prior to entry into an IVF cycle to detect abnormal vaginal flora, or by culturing the follicular fluid collected at the time of trans-vaginal oocyte retrieval for the presence of microorganisms. Treatment with antimicrobials may increase IVF treatment success rates.

## Supporting Information

Table S1 **Table S1 describes the microorganisms cultivated from paired follicular fluid and vaginal swab specimens collected from women enrolled in this study.** The microbial data is stratified by the cause of infertility.
(DOCX)
