# Supplementary material for: Microorganisms within Human Follicular Fluid: Effects on IVF
Source: PLoS One. 2013 Mar 12;8(3):e59062. doi: 10.1371/journal.pone.0059062 (PMC3595219; doi:10.1371/journal.pone.0059062)
Supplement: Table S1 — describes the microorganisms cultivated from paired follicular fluid and vaginal swab specimens collected from women enrolled in this study. The microbial data is stratified by the cause of infertility. (DOCX) [file pone.0059062.s001.docx]

Supplementary Table S1 Microorganisms detected in the follicular fluid and vaginal swabs of women with various causes of infertility

| **Colonized Contaminated Male factor Endometriosis Polycystic Genital Idiopathic**  **(fertile women) ovary infection**  **syndrome** | | | | | | | | | | | | | | | | | | | | | |
| --- | --- | --- | --- | --- | --- | --- | --- | --- | --- | --- | --- | --- | --- | --- | --- | --- | --- | --- | --- | --- | --- |
| **Microbial species** | Left  Ovary  (L) | Right  Ovary  (R) | Vagina  (V) | Left | Right | Vagina | L | R | V | L | R | V | L | R | V | L | R | V | L | R | V |
| ***Actinomyces* species** | A +  B | + | - | + | + | + | 35  31 | 35  26 | 5  5 | -  - | -  - | -  - | -  - | -  - | -  - | 28  16 | 14  9 | 5  4 | 61  15 | 48  13 | -  - |
| ***A. israelii*** | - | + | - | - | + | + | -  - | -  - | -  - | -  - | -  - | -  - | -  - | -  - | -  - | 3  - | -  - | 4  - | -  - | -  - | -  - |
| ***A. meyeri*** | - | - | + | + | + | + | -  - | -  - | 1  1 | -  - | -  - | -  - | -  - | -  - | -  - | -  2 | -  - | -  - | -  - | -  - | 2  2 |

| *A. naeslundii* | + | - | + | + | + | + | 4  - | 2  - | 8  8 | -  2 | -  - | 16  4 | -  - | -  - | -  - | -  - | -  - | 6  6 | -  - | 4  - | -  - |
| --- | --- | --- | --- | --- | --- | --- | --- | --- | --- | --- | --- | --- | --- | --- | --- | --- | --- | --- | --- | --- | --- |
| *Bacteroides* spp. | - | + | + | - | + | + | -  - | -  1 | -  - | -  - | 5  3 | -  - | -  - | 3  - | -  - | -  - | 12  3 | -  - | -  - | -  - | 7  1 |
| *Bacteroides fragilis* | + | + | + | + | + | + | 1  - | 3  - | -  - | -  - | 6  3 | -  - | -  - | -  - | 6  - | -  1 | 6  3 | 6  3 | 22  3 | -  - | -  - |
| *Bifidobacterium* spp. 1 | + | + | + | + | + | + | 26  - | 14  5 | 52  19 | 1  - | 1  - | 58  41 | 28  12 | 13  - | 32  10 | 7  4 | 21  15 | 15  18 | 12  14 | 7  8 | 83  12 |
| *Bifidobacterium* spp. 2 | - | + | + | - | + | + | -  - | -  - | 17  12 | -  - | 1  - | -  - | -  - | -  - | 51  33 | -  - | 1  3 | 19  7 | -  - | 1  - | 61  8 |
| *Candida albicans* | + | + | + | - | + | + | -  - | -  - | 35  10 | -  - | -  - | 24  6 | -  1 | -  - | 12  - | -  - | 12  8 | 10  - | -  - | -  - | -  - |
| *C. glabrata* | + | + | + | + | + | + | -  - | -  - | 7  2 | -  - | 8  - | 12  10 | -  - | -  - | 6  - | -  - | -  - | 15  4 | 22  3 | 6  7 | 64  8 |
| *C. parapsilosis* | + | + | - | + | + | + | -  - | -  - | -  - | -  11 | -  - | -  - | -  - | -  - | -  - | 1  - | 4  3 | -  - | -  - | -  - | 5  - |

| *Clostridium butyricum* | - | - | + | + | - | + | -  - | -  - | 15  - | -  - | -  - | -  - | -  - | -  - | 2  1 | -  - | -  - | 5  5 | 2  - | -  - | 29  10 |
| --- | --- | --- | --- | --- | --- | --- | --- | --- | --- | --- | --- | --- | --- | --- | --- | --- | --- | --- | --- | --- | --- |
| *Clostridium* spp. | - | - | + | + | - | + | 1  - | -  - | -  - | -  - | -  - | -  - | -  - | -  - | 2  4 | -  - | -  - | -  - | -  - | -  - | -  - |
| *C. ramosum* | - | - | + | - | - | + | -  - | -  - | 4  4 | -  - | -  - | -  - | -  - | -  - | -  - | -  - | -  - | 9  - | -  - | -  - | -  - |
| *Corynebacterium* spp. | - | + | + | - | - | + | -  - | -  - | 8  5 | -  - | -  - | -  - | -  - | -  1 | 10  2 | -  - | -  - | 5  13 | -  - | -  - | -  - |
| *C. auromucosum* | - | - | - | - | - | + | -  - | -  - | -  - | -  - | -  - | -  - | -  - | -  - | -  - | -  - | -  - | -  - | -  - | -  - | 3  - |
| *Escherichia coli* | + | + | + | - | + | + | -  - | -  - | 15  4 | -  - | -  - | -  11 | -  - | -  - | 7  2 | -  1 | 6  4 | 21  7 | -  - | 1  - | 22  3 |
| *Enterococcus faecalis* | - | + | + | - | - | + | -  - | -  - | 6  6 | -  - | -  - | 10  - | -  - | -  24 | 11  10 | -  - | -  - | 7  2 | -  - | -  - | -  - |
| *Egghertella lenta* | - | - | + | - | - | + | -  - | -  - | 4  1 | -  - | -  - | -  - | -  - | -  - | 2  1 | -  - | -  - | 8  - | -  - | -  - | 12  - |

| *Fusobacterium* spp. | - | - | - | + | - | + | 1  - | -  - | -  - | -  - | -  - | 10  - | -  - | -  - | -  - | -  - | -  - | -  - | -  - | -  - | -  - |
| --- | --- | --- | --- | --- | --- | --- | --- | --- | --- | --- | --- | --- | --- | --- | --- | --- | --- | --- | --- | --- | --- |
| *Gemella* spp. | - | - | + | - | - | + | -  - | -  - | -  - | -  - | -  - | -  - | -  - | -  - | -  - | -  - | -  - | 17  5 | -  - | -  - | 3  - |
| *Klebsiella* spp*.* | - | - | + | - | - | + | -  - | -  - | -  - | -  - | -  - | -  - | -  - | -  - | 2  1 | -  - | -  - | 1  2 | -  - | -  - | -  - |
| *Lactobacillus* spp. | + | + | + | + | + | + | 16  31 | 12  11 | 67  37 | 7  6 | 16  7 | 81  36 | 32  25 | 13  1 | 65  7 | 8  4 | 5  3 | 42  15 | 66  13 | 33  13 | 125  9 |
| *L. crispatus* | + | + | + | + | + | + | 18  10 | 5  5 | 51  21 | 19  12 | 3  10 | 24  4 | 4  1 | -  - | 31  30 | 20  13 | 7  4 | 52  5 | 1  - | -  - | 70  7 |
| *L. gasseri* | + | - | + | + | + | + | 3  - | -  - | 23  2 | 2  6 | 2  - | 3  3 | 1  - | -  - | 9  1 | 3  1 | -  - | 9  - | -  - | -  - | 15  2 |
| *L. iners* | - | - | - | - | + | - | -  - | -  - | -  - | -  - | -  - | -  - | -  - | 3  - | -  - | -  - | -  - | -  - | -  - | -  - | -  - |

| *L. jensenii* | + | + | + | + | - | + | 14  - | -  - | 42  - | 9  11 | -  10 | 17  3 | -  - | -  - | 29  1 | 3  1 | -  2 | 14  1 | 4  - | -  - | 24  4 |
| --- | --- | --- | --- | --- | --- | --- | --- | --- | --- | --- | --- | --- | --- | --- | --- | --- | --- | --- | --- | --- | --- |
| *Propionibacterium* spp*.* | + | - | - | - | - | + | -  - | -  - | 16  - | -  - | -  - | -  - | -  1 | -  - | -  - | -  - | -  - | -  - | -  - | -  - | -  - |
| *P. acnes* | + | + | + | + | + | + | 22  21 | 33  31 | 35  13 | -  11 | -  - | 2  3 | -  - | -  - | 3  - | -  1 | -  1 | 2  2 | 60  13 | 85  25 | 54  2 |
| *P. avidum* | - | - | + | - | - | + | -  - | -  - | 10  1 | -  - | -  - | -  - | -  - | -  - | 9  - | -  - | -  - | -  - | -  - | -  - | 8  - |
| *P. granulosum* | + | + | - | + | + | + | 1  5 | 1  5 | 4  - | -  - | -  - | -  - | -  - | -  - | -  - | -  - | -  - | 10  - | -  - | -  - | 11  - |
| *P. propionicus* | + | + | + | + | - | + | -  - | -  - | 6  3 | -  - | -  - | 2  3 | -  - | -  - | 2  4 | -  1 | -  - | -  - | 6  7 | -  2 | -  - |
| *Prevotella disiens* | - | - | + | - | - | + | -  - | -  - | 5  1 | -  - | -  - | -  - | -  - | -  - | -  - | -  - | -  - | 15  - | -  - | -  - | -  - |

| *P. melanogenicus* | - | - | + | - | - | + | -  - | -  - | 4  1 | -  - | -  - | -  - | -  - | -  - | -  - | -  - | -  - | 2  3 | -  - | -  - | 4  2 |
| --- | --- | --- | --- | --- | --- | --- | --- | --- | --- | --- | --- | --- | --- | --- | --- | --- | --- | --- | --- | --- | --- |
| *Peptinophilus asaccharolyticus* | + | - | + | + | + | + | 1  - | -  - | 10  3 | -  - | -  - | -  - | -  - | -  - | 2  4 | -  1 | 6  - | -  - | -  - | -  - | -  - |
| *Peptostreptococcus* spp. | + | + | + | + | + | + | 2  - | -  - | 1  1 | -  - | -  - | -  - | 13  1 | -  - | -  - | -  1 | -  5 | -  2 | -  - | 22  3 | -  - |
| *Staphylococcus* spp. | + | + | + | - | + | + | -  - | -  - | 18  6 | -  11 | 2  10 | 6  10 | -  - | -  - | 15  3 | -  1 | -  - | 15  12 | -  - | 7  - | 17  1 |
| *S. aureus* | + | - | + | + | + | + | -  - | -  - | 29  10 | -  - | -  - | 30  15 | -  - | -  - | 3  3 | 1  1 | 1  - | 21  8 | 22  - | -  - | 50  7 |
| *S. epidermidis* | + | + | + | + | + | + | -  - | 2  - | 41  11 | -  - | -  - | 36  24 | -  - | -  - | -  - | 3  1 | 9  6 | 3  2 | 6  7 | 6  7 | 32  6 |
| *S. lugdunensis* | + | - | + | + | + | + | -  - | -  - | 22  8 | -  - | -  - | 28  13 | -  - | -  - | 8  4 | 6  4 | 7  4 | 6  3 | -  - | 1  - | 8  2 |
| *Streptococcus* spp. | + | + | - | + | + | + | -  - | -  - | 4  - | -  - | -  - | -  - | -  - | -  - | 6  - | 6  5 | 6  4 | -  - | -  - | -  - | -  - |

| *S. agalactiae* | + | + | + | + | + | + | 10  - | 1  - | 31  16 | -  - | -  - | 52  38 | 15  13 | -  - | 20  15 | 1  - | 5  3 | 17  9 | -  - | -  - | 34  8 |
| --- | --- | --- | --- | --- | --- | --- | --- | --- | --- | --- | --- | --- | --- | --- | --- | --- | --- | --- | --- | --- | --- |
| *S. intermedius* | + | + | + | + | + | + | 1  - | 1  - | 39  12 | -  - | -  - | 12  10 | -  - | -  - | 15  3 | -  - | 2  - | 9  3 | -  1 | 6  7 | 11  6 |
| *S. viridans* | - | - | - | + | - | + | 2  - | -  - | -  - | -  - | -  - | -  - | -  - | -  - | -  - | -  - | -  - | -  - | -  - | -  - | 15  - |
| *Veilonella* spp. | - | + | + | + | + | + | 1  - | -  - | 1  1 | -  - | -  - | -  - | -  - | 15  12 | 2  1 | -  - | -  - | 3  - | -  - | -  7 | -  - |
| *Unknown A* | - | - | - | - | - | + | -  - | -  - | -  - | -  - | -  - | -  - | -  - | -  - | -  - | -  - | -  - | 2  - | -  - | -  - | 5  - |
| *Unknown B* | - | - | + | - | - | + | -  - | -  - | 2  - | -  - | -  - | -  - | -  - | -  - | 7  2 | -  - | -  - | -  - | -  - | -  - | -  - |
| **Total number 257 193 862 108 87 657 147 85 511 149 204 516 360 319 874**  **of isolates** | | | | | | | | | | | | | | | | | | | | | |

+ present, - absent; A (top line of each species) present as ‘contaminants’, B (bottom line of each species) present as ‘colonizers’, numbers represent the number of women each species was detected in left and right follicular fluids and vaginal swabs.
